# Transient ciliochoroidal detachment after 360-degree suture trabeculotomy ab interno for open-angle glaucoma: 12-month follow-up

**DOI:** 10.1038/s41433-019-0375-5

**Published:** 2019-02-21

**Authors:** Tomoki Sato, Takahiro Kawaji, Akira Hirata

**Affiliations:** 1 Sato Eye and Internal Medicine Clinic, 4160-270 Arao, Arao City, Kumamoto, 864-0041 Japan; 2 Hayashi Eye Hospital, 4-23-35 Hakataekimae, Hakata-ku, Fukuoka, 812-0011 Japan

**Keywords:** Outcomes research, Glaucoma

## Abstract

**Objectives:**

To investigate the effects of ciliochoroidal detachment (CCD) after 360-degree suture trabeculotomy ab interno (360S-LOT ab interno) on intraocular pressure (IOP) and postoperative complications during a 12-month follow-up.

**Methods:**

We prospectively examined 44 eyes of 44 patients for 12 months after 360S-LOT ab interno surgery. Inclusion criteria were open-angle glaucoma including primary open-angle glaucoma, normal tension glaucoma, and exfoliation glaucoma without previous glaucoma surgeries. CCD was detected by swept-source anterior segment optical coherence tomography. Outcome measures were the course of IOP, the number of medications, and the postoperative complications of the CCD group compared with those of the non-CCD group.

**Results:**

CCD appeared in 21 eyes (47.7%) within postoperative day 7 and disappeared within postoperative month 1 in 19 of 21 eyes. Although the IOP on postoperative day 1 in the CCD group (11.9 ± 7.7 mmHg) was significantly lower than that in the non-CCD group (19.2 ± 12.8 mmHg) (*P* *=* 0.020), the difference in the postoperative IOP between the groups decreased with time. No significant difference was seen in the number of medications and postoperative complications.

**Conclusions:**

CCD occurred in approximately half of patients after 360S-LOT ab interno and may have the transient effect of lowering the IOP immediately after surgery. Postoperative CCD had no effect on the later IOP, the number of medications and postoperative complications throughout 12-month of follow-up.

## Introduction

The mechanism of action of a trabeculotomy involves a reduction in abnormal outflow resistance via removal of the inner wall of the Schlemm’s canal and the trabecular meshwork [1, 2]. Several new ab interno trabeculotomy devices and procedures, such as the Trabectome (NeoMedix, Tustin, CA) [3], iStent (Glaukos, Laguna Hills, CA) [4], microhook ab interno trabeculotomy [5], gonioscopy-assisted transluminal trabeculotomy [6], and suture trabeculotomy ab interno [7], which do not require incision of the conjunctiva and the sclera, have emerged as low-risk surgical approaches for open-angle glaucoma (OAG).

Although these procedures can achieve a modest reduction in intraocular pressure (IOP), they cannot lower the IOP beyond the episcleral venous pressure, which ranges from 7.6 to 11.4 mm Hg in seated individuals [8]. However, ~1% of patients reportedly developed early transient hypotony (< 5 mm Hg) on the first postoperative day after the Trabectome was used [9, 10]. The reason for this unexpectedly low IOP in the early postoperative period and the effect of this low IOP on the outcome of ab interno trabeculotomy have not yet been fully investigated. Akagi et al. demonstrated the presence of ciliochoroidal detachment (CCD) in 42% cases by using anterior segment optical coherence tomography (AS-OCT), and the CCD was associated with low IOP immediately after Trabectome surgery during 3-month follow-up period [11]. These results suggested that CCD may result partly from the transient increase in uveoscleral aqueous outflow via the trabeculotomy site. Several previous studies also reported that postoperative CCD after some types of surgery (e.g., deep sclerectomy [12], trabeculectomy [13, 14], and ab interno microstent implantation [15]) is due to an acceleration of the uveoscleral outflow of aqueous humor. However, because the follow-up period was short in these studies, approximately <3 months, whether CCD in the early postoperative period affected mid- or long-term results is still unclear.

We previously reported a reduction of the IOP value to approximately the middle-teen after 360-degree suture trabeculotomy ab interno (360S-LOT ab interno). In this surgery, we used a 360-degree incision for the inner wall of Schlemm’s canal and the trabecular meshwork. We also observed an IOP value of <10 mm Hg on the first postoperative day in a few cases [7]. Postoperative CCD may have occurred more frequently and affected subsequent outcomes in eyes after a 360-degree incision of Schlemm’s canal via 360S-LOT ab interno than after the approximately 120-degree incision via the Trabectome.

In the present study, we prospectively investigated the presence and extent of CCD by using high-resolution swept-source AS-OCT before and after 360S-LOT ab interno, and we studied the effects of postoperative CCD on postoperative IOP during a 12-month follow-up period.

## Subjects and methods

### Subjects

This prospective, open-label, single-center, observational clinical study was performed at the Sato Eye and Internal Medicine Clinic (Kumamoto, Japan) between November 2015 and January 2017. The study protocol adhered to the tenets of the Declaration of Helsinki, was approved by the Institutional Review Board and the Ethics Committee of Sato Eye and Internal Medicine Clinic, and was registered with the University Hospital Medical Information Network Clinical Trials Registry of Japan with the registration number UMIN000021128. Before surgery, informed written consent was obtained from all patients.

Patients were considered for enrollment in this study if they had OAG, including primary open-angle glaucoma, normal tension glaucoma, and exfoliation glaucoma without previous glaucoma surgeries, that was refractory to medical therapy or had OAG requiring medical therapy with a visually significant cataract. A cataract was considered visually significant if the patient complained of glare or halos and the best-corrected visual acuity (BCVA) was 20/20 or worse. Primary open-angle glaucoma was diagnosed if IOP was >21 mm Hg, the angle was open (Shaffer grade 3 or 4), and optic neuropathy with matching visual field changes had occurred. Normal tension glaucoma was diagnosed by using the same criteria, but if IOP was ≤21 mm Hg. Exfoliation glaucoma was diagnosed if exfoliation material had adhered to the lens surface or the iris-pupil margin, the angle was wide open in the presence of dense pigmentation, Sampaolesi’s line was present, and optic neuropathy with matching visual field loss had occurred. Subjects were excluded from this study if they had any of the following: other types of glaucoma; a history of previous intraocular surgery; or an ocular condition that would compromise their safety or interfere with study testing.

All procedures were performed by one surgeon (T.S.) who used the surgical procedure described herein. If a patient underwent bilateral surgery, only the first eye operated on was included in the analysis.

### 360S-LOT ab interno procedure

This procedure was previously described in full [7], and a modified technique for Schlemm’s canal incision was used for this study. Briefly, a 1.7-mm temporal corneal incision was made, and Schlemm’s canal was incised at 15 degrees on the nasal side by using a microhook needle (HS-2167; Handaya, Tokyo, Japan) instead of the Trabectome, which was used in the original paper [7]. The rounded tip of a 5-0 nylon suture was then inserted into Schlemm’s canal by using a 23-gauge disposable grasping forceps (DSP forceps; Alcon, Tokyo, Japan). After the suture tip was passed around the circumference of Schlemm’s canal, the suture was pulled out through the same opening and then a 360-degree incision was made. If necessary, standard phacoemulsification with intraocular lens implantation was performed after the 360S-LOT ab interno procedure through the same incision or a newly created upper corneal incision. At the end of surgery, we confirmed that no wound leakage occurred and that the IOP value with the patient in the supine position was 18 mm Hg or more by using Icare (M.E. Technica, Tokyo, Japan). In addition, we measured the IOP at 30 min after surgery by again using Icare.

### Examinations

After patients gave informed consent, they were subjected to screening, with all patients undergoing slit-lamp biomicroscopy, indirect ophthalmoscopy, manifest refraction, measurement of IOP, and determination of BCVA by means of a conventional Landolt ring chart. Decimal visual acuities were converted to the logarithm of the minimum angle of resolution units for the analyses. We obtained three separate IOP measurements within 1 month before surgery and used the average of these measurements as the IOP baseline. We examined patients at 1, 2, and 3 days after surgery and then 1 to 2 weeks thereafter for 1 month, every month until 6 months, and then every 2 or 3 months until 12 months. We measured the IOP at every study visit. Subjects were given anti-glaucoma medications after surgery if the IOP value was higher than the desired range (<15 mm Hg). All patients underwent swept-source AS-OCT examination (CASIA2; Tomey, Tokyo, Japan) preoperatively and on postoperative days 1, 3, and 7. In addition, if the AS-OCT images showed any CCD on postoperative day 7, an AS-OCT examination was performed every 1 month until no CCD was seen on AS-OCT images.

### Outcome measures

The outcome measures were the following: the occurrence of CCD within 7 days after 360S-LOT ab interno, the course of IOP, the number of medications and the postoperative complications in the CCD group and the non-CCD group. We also analyzed the factors associated with the postoperative IOP levels, with the independent variables being age, sex, diagnosis, axial length, central corneal thickness, visual field mean deviation, surgical procedure, preoperative IOP, preoperative glaucoma medication score, and CCD within postoperative day 7.

### AS-OCT measurement

**Fig. 1:**
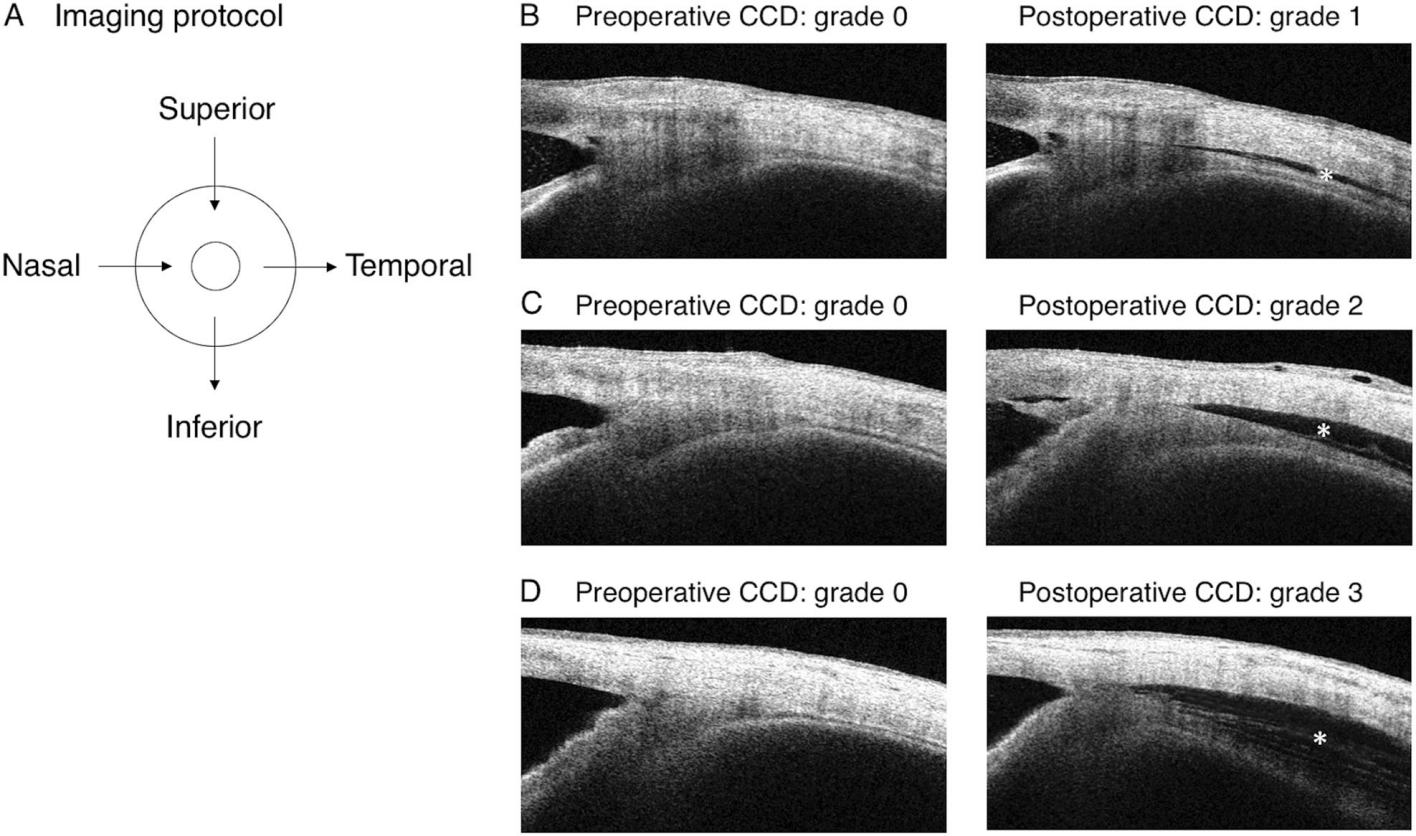
Imaging protocol and preoperative and postoperative images obtained by AS-OCT. **a** Imaging was performed independently in each of the four quadrants (superior, inferior, nasal, and temporal). **b**–**d** No preoperative CCD was observed on the left. Detachments were observed postoperatively on the right at the same location. The asterisks indicate CCDs

The patient, in a seated position, was asked to look to the opposite side during imaging. With care taken not to press on the globe, imaging was performed for each of the four independent quadrants (superior, inferior, nasal, and temporal) (Fig. 1a). All participants underwent AS-OCT imaging in a well-lit room. CCD severity was classified on the basis of the maximum CCD among AS-OCT images, as previously reported [8, 11]. Classification included grade 0 (no sign of CCD) (Fig. 1b–d, left), grade 1 (slit-like, with CCD less than half of the ciliary body thickness) (Fig. 1b, right), grade 2 (band-like, with CCD at least half of the ciliary body thickness) (Fig. 1c, right), and grade 3 (obvious, with CCD greater than the ciliary body thickness) (Fig. 1d, right). The eyes with CCD (grades 1–3) and eyes without CCD (grade 0) within postoperative day 7 comprised the CCD group and the non-CCD group, respectively. Variables analyzed included age, sex, glaucoma type, axial length, central corneal thickness, visual field mean deviation, phacoemulsification combination procedure, preoperative IOP, number of anti-glaucoma medications, and postoperative CCD+/− within postoperative day 7.

### Safety

Evaluation of safety included postoperative complications. In terms of postoperative complications, an IOP value of ≥ 30 mm Hg within 1 month of the surgery and a day-to-day increase in IOP of ≥ 5 mm Hg were defined as IOP spikes. Hypotony was defined as IOP ≤ 5 mm Hg.

### Statistical analyses

JMP version 12 (SAS Institute, Inc., Cary, NC, USA) was used for data analysis. Because of the small sample size in this study, continuous variables were expressed as the mean ± standard deviation and were then analyzed by means of the Mann–Whitney nonparametric test. Categorical variables were compared by using Fisher’s exact test. To determine the factors associated with postoperative IOP levels, we performed a stepwise multiple regression analysis. The statistical significance level was set at *P* < 0.05.

## Results

The 360S-LOT ab interno procedure was performed on 60 eyes between November 2015 and January 2017. Twenty-one patients were excluded because they did not have OAG (11 eyes) or had past glaucoma surgeries (two eyes). In addition, 3 of 47 subjects (6.4%) were not present at the 12-month visit because of poor health and were excluded from analyses. Therefore, 44 eyes of 44 subjects (20 males, 73.4 ± 9.2 years old) were ultimately included in the 12-month analyses (Table 1). We used the 360S-LOT ab interno procedure alone for nine eyes (20.5%) and in combination with phacoemulsification for 35 eyes (79.5%). We achieved a complete 360-degree Schlemm’s canal incision in 42 of 44 eyes (95.5%); for the other two eyes we used a 270- or 330-degree incision for Schlemm’s canal.

**Table 1:** Clinical characteristics of the study patients

Variable	Non-CCD group, grade 0	CCD group, grades 1–3	95% CI of the difference	Effect size *γ*	Power, 1−*β* coefficient	CCD grade			CCD range			
						Grade 1	Grade 2	Grade 3	1 quadrant	2 quadrants	3 quadrants	4 quadrants
	(*n* = 23)	(*n* = 21)				(*n* = 12)	(*n* = 3)	(*n* = 6)	(*n* = 4)	(*n* = 2)	(*n* = 3)	(*n* = 12)
Age, mean ± SD, years	76.9 ± 10.6	73.7 ± 10.1	−11.6 to 1.8	0.22	0.31	75.6 ± 10.5	72.3 ± 8.6	70.5 ± 10.8	82.0 ± 6.2	74.5 ± 13.4	64.0 ± 12.3	73.2 ± 9.0
Sex, Male/female, No	15/8	12/9	NA	NA	NA	6/6	2/1	4/2	3/1	2/0	0/3	7/5
Diagnosis, No												
Primary open-angle glaucoma	9	11	NA	NA	NA	7	1	3	3	1	2	5
Normal tension glaucoma	7	6	NA	NA	NA	3	0	3	1	1	0	4
Exfoliation glaucoma	7	4	NA	NA	NA	2	2	0	0	0	1	3
Axial length, mean ± SD, mm	24.2 ± 1.8	24.2 ± 1.4	−1.4 to 0.7	0.10	0.099	24.0 ± 1.4	23.8 ± 1.1	24.6 ± 1.7	23.8 ± 0.5	26.0 ± 1.3	24.4 ± 1.4	23.9 ± 1.5
Central corneal thickness, mean ± SD, μm	524.5 ± 21.0	509.1 ± 41.4	−31.4 to 11.7	0.14	0.14	510.8 ± 42.5	476.3 ± 58.8	522.2 ± 25.6	502 ± 47.4	509.0 ± 8.5	522.7 ± 55.9	508.1 ± 43.2
Visual field mean deviation, mean ± SD, dB	−8.7 ± 6.4	−12.7 ± 11.0	−9.9 to 2.1	0.25	0.20	−8.2 ± 8.9	−25.8 ± 4.9	−15.5 ± 12.2	−14.5 ± 11.9	−8.6 ± 11.9	−2.5 ± 1.7	−15.5 ± 11.8
Surgical procedure, No												
Combined	18	17	NA	NA	NA	10	2	5	4	2	1	10
Single	5	4	NA	NA	NA	2	1	1	0	0	2	2
Preoperative lens status, No.												
Phakia	18	21	NA	NA	NA	12	3	6	4	2	3	12
Intraocular lens	5	0	NA	NA	NA	0	0	0	0	0	0	0
IOP, mean ± SD, mm Hg												
Preoperative	17.9 ± 5.3	17.1 ± 7.1	−2.9 to 4.7	0.072	0.074	17.4 ± 1.8	17.9 ± 3.7	16.0 ± 2.6	15.1 ± 1.8	13.9 ± 0.2	26.0 ± 16.9	16.0 ± 3.5
Postoperative day 1	19.2 ± 12.8	11.9 ± 7.7	0.8 to 13.8	0.25	0.60	14.0 ± 3.1	7.7 ± 6.2	9.8 ± 4.4	19.3 ± 14.1	10.5 ± 0.7	12.3 ± 6.1	9.6 ± 4.6
Postoperative day 3	15.3 ± 6.4	12.4 ± 8.1	−1.5 to 7.3	0.20	0.25	15.1 ± 2.0	7.7 ± 4.1	9.3 ± 2.9	15.5 ± 11.8	18.0 ± 5.7	12.0 ± 4.0	10.5 ± 7.8
Postoperative day 7	18.0 ± 9.3	13.8 ± 6.5	−0.8 to 9.1	0.38	0.38	15.4 ± 2.4	13.0 ± 4.7	11.0 ± 3.3	16.5 ± 8.2	17.0 ± 1.4	15.0 ± 7.0	12.1 ± 6.5
Postoperative month 1	12.1 ± 3.5	15.7 ± 9.1	−7.7 to 0.5	0.26	0.12	15.7 ± 2.0	12.0 ± 3.9	17.7 ± 2.8	11.0 ± 2.9	21.0 ± 11.3	20.7 ± 9.7	15.2 ± 9.9
Postoperative month 3	12.8 ± 4.4	13.0 ± 3.8	−2.7 to 2.5	0.016	0.050	13.1 ± 1.2	14.0 ± 2.4	12.0 ± 1.9	13.0 ± 3.5	11.0 ± 1.4	15.7 ± 7.2	12.5 ± 3.2
Postoperative month 6	12.4 ± 2.6	12.8 ± 3.6	−2.3 to 1.0	0.056	0.064	13.6 ± 0.9	12.7 ± 1.8	10.8 ± 1.4	13.0 ± 2.0	12.5 ± 0.7	17.0 ± 7.0	11.5 ± 2.5
Postoperative month 12	13.5 ± 3.9	13.0 ± 3.1	−1.6 to 2.7	0.075	0.076	13.9 ± 1.7	14.3 ± 2.0	10.5 ± 1.4	12.5 ± 1.0	14.5 ± 2.1	16.3 ± 2.9	12.1 ± 3.3
Glaucoma medication score, mean ± SD												
Preoperative	1.8 ± 1.8	2.9 ± 1.5	−2.1 to −0.1	0.32	0.56	2.8 ± 0.5	4.3 ± 0.9	2.5 ± 0.7	2.3 ± 0.4	3.5 ± 1.2	3.0 ± 1.0	3.0 ± 0.5
Postoperative month 12	1.0 ± 1.3	0.8 ± 1.1	−0.5 to 0.4	0.080	0.080	1.0 ± 0.4	0.7 ± 0.7	0.5 ± 0.5	0.8 ± 0.6	0.5 ± 0.6	2.3 ± 0.7	0.5 ± 0.3
Visual acuity, mean ± SD, logMAR												
Preoperative	0.26 ± 0.21	0.37 ± 0.48	−0.32 to 0.11	0.054	0.16	0.29 ± 0.33	0.38 ± 0.40	0.53 ± 0.75	0.46 ± 0.42	0.55 ± 0.21	0.073 ± 0.20	0.38 ± 0.57
Postoperative month 12	0.084 ± 0.22	0.050 ± 0.20	−0.094 to 0.16	0.017	0.083	−0.01 ± 0.10	0.032 ± 0.044	0.037 ± 0.082	−0.022 ± 0.12	0.07 ± 0.21	−0.027 ± 0.05	0.09 ± 0.24
Postoperative compications, No												
Postoperative hypotony	1	4	NA	NA	NA	0	1	3	0	0	0	4
Postoperative hyphema	4	7	NA	NA	NA	1	1	5	0	0	1	6
Postoperative IOP spike	9	5	NA	NA	NA	2	1	2	1	0	0	4

*CCD* ciliochoroidal detachment, *IOP* intraocular pressure, *NA* not applicable

### Outcomes

**Fig. 2:**
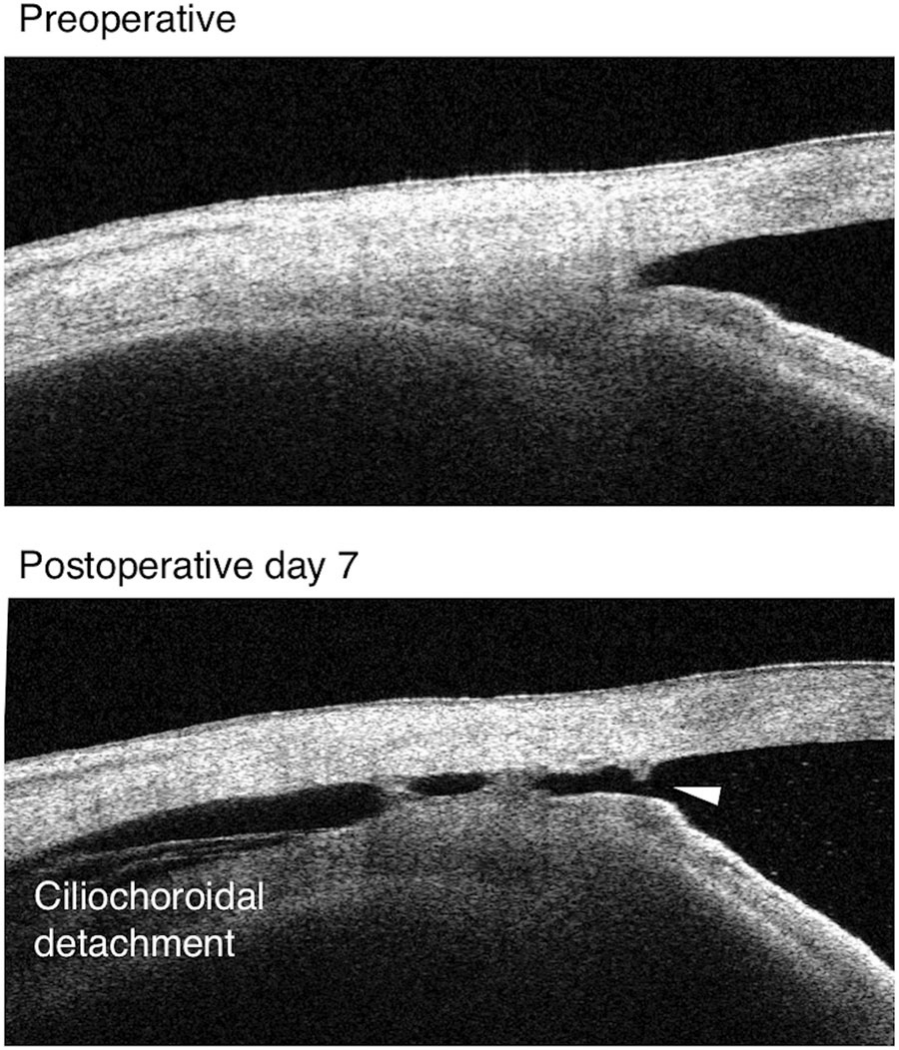
The connection between CCD and the anterior chamber. The white arrowhead shows that the CCD was connected to the anterior chamber

**Fig. 3:**
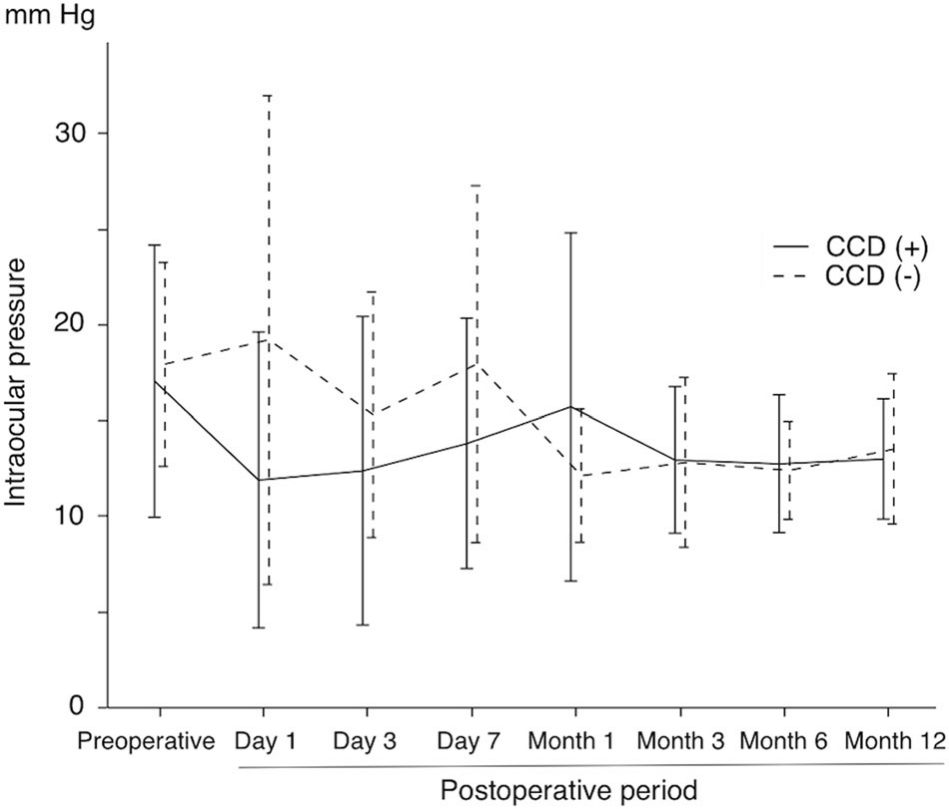
Time course of IOP in eyes with CCD (CCD group) and without CCD (non-CCD group)

The mean IOP value in all eyes was significantly reduced from 17.5 ± 6.2 mm Hg to 13.3 ± 3.5 mm Hg (24.0% reduction; *P* = 0.003), and the number of medications was also significantly reduced from 2.3 ± 1.7 to 0.9 ± 1.2 (*P* = 0.001). The 44 eyes in the study showed no obvious CCD before surgery. Within postoperative day 7, 21 eyes (47.7%) had grades 1–3 CCD (CCD group), whereas 23 eyes (52.3%) had no CCD (non-CCD group) (Table 1 and Fig. 1). The IOP value at the end of surgery was 31.5 ± 8.5 mm Hg in the CCD group and 29.8 ± 9.5 mm Hg in the non-CCD group, according to Icare. IOP at 30 min after surgery was 12.0 ± 4.7 mm Hg in the CCD group and 13.0 ± 4.2 mm Hg in the non-CCD group, without wound leakage in both groups. Of the 21 CCD eyes, CCD was first detected in 14 eyes on postoperative day 1, five eyes on postoperative day 3, and two eyes on postoperative day 7. CCD disappeared within postoperative day 7 in 9 eyes, from postoperative day 7 to month 1 in 10 eyes, and from postoperative month 2 to month 3 in two eyes. Of the two eyes with prolonged CCD: one had grade 3 and all quadrants affected; the other had grade 1 and one quadrant affected. In the CCD group, 12 eyes (57.1%) had CCD in all quadrants, 3 eyes (14.3%) had CCD in three quadrants, 2 eyes (9.5%) had CCD in two quadrants, and 4 eyes (19.0%) had CCD in one quadrant. Also, the maximum CCD grade was grade 1 in 13 eyes (61.9%), grade 2 in 3 eyes (14.3%), and grade 3 in 5 eyes (23.8%). In addition, six eyes (28.6%) demonstrated a connection between the anterior chamber and CCD (Fig. 2). The root of the iris was also seen in five eyes during Schlemm’s canal incision during use of the microhook needle. The preoperative glaucoma medication score was lower in the non-CCD group than in the CCD group (Table 1). Although the postoperative day 1 IOP value was lower in the CCD group than in the non-CCD group, the postoperative IOP value through postoperative month 12 were similar in both the CCD and non-CCD groups (Fig. 3).

With regard to postoperative complications, we observed hypotony in one eye in the non-CCD group and in four eyes in the CCD group; hyphema in four and seven eyes, respectively; and an IOP spike in nine and five eyes, respectively. No significant differences in the presence of these complications were seen in both groups (*χ*^2^ test, *P* *=* 0.12, 0.22, 0.27, respectively).

Using a stepwise multiple regression analysis, we found that higher CCD grades within postoperative day 7 were associated with lower IOP values on postoperative days 1, 3, and 7; wider CCD quadrants within postoperative day 7 were associated with lower IOP values on postoperative days 1 and 7 (Table 2). Table 2 summarizes other associations with postoperative CCD and additional factors. The combined procedure and a preoperative lower IOP value were associated with lower IOP values at postoperative month 12.

**Table 2:** Postoperative IOP as associated with postoperative CCD, medications, surgical procedure, and other variables

	IOP at postoperative day 1		IOP at postoperative day 3		IOP at postoperative day 7		IOP at postoperative month 1		IOP at postoperative month 3		IOP at postoperative month 6		IOP at postoperative month 12	
Variable	*β* Coefficient (95% CI)	*P* Value	*β* Coefficient (95% CI)	*P* Value	*β* Coefficient (95% CI)	*P* Value	*β* Coefficient (95% CI)	*P* Value	*β* Coefficient (95% CI)	*P* Value	*β* Coefficient (95% CI)	*P* Value	*β* Coefficient (95% CI)	*P* Value
Age, years	NA	0.27	NA	0.51	NA	0.71	−0.307 (−0.491 to −0.123)	0.002	NA	0.085	0.048 (−0.222 to −0.029)	0.012	NA	0.51
Male sex	NA	0.26	NA	0.66	NA	0.089	NA	0.72	NA	0.82	NA	0.48	NA	0.42
Axial length, mm	NA	0.66	NA	0.98	NA	0.15	1.457 (0.168 to 2.747)	0.028	NA	0.77	NA	0.83	NA	0.55
Central corneal thickness, μm	0.334 (0.041 to 0.574)	0.027	0.367 (0.079 to 0.599)	0.014	NA	0.18	NA	0.46	0.043 (0.006 to 0.080)	0.024	NA	0.083	NA	0.10
Visual field mean deviation, dB	NA	0.68	NA	0.87	NA	0.88	NA	0.36	NA	0.51	NA	0.17	NA	0.21
Combined surgical procedure	NA	0.83	NA	0.73	NA	0.22	NA	0.055	−2.776 (−4.164 to −1.388)	<0.001	−2.195 (−3.206 to −1.183)	<0.001	−2.342 (−3.477 to −1.209)	<0.001
Preoperative IOP, mm Hg	NA	0.35	NA	0.51	NA	0.18	NA	0.055	0.206 (0.008 to 0.405)	0.042	0.195 (0.049 to 0.342)	0.010	0.253 (0.009 to 0.413)	0.003
Preoperative glaucoma medication	NA	0.35	NA	0.65	NA	0.52	1.782 (0.652 to 2.913)	0.003	NA	0.23	NA	0.12	NA	0.11
CCD, grades 0–3	−0.343 (−0.581 to −0.051)	0.023	−0.317 (−0.561 to −0.022)	0.036	−0.302 (−0.549 to −0.005)	0.047	NA	0.095	NA	0.88	NA	0.50	NA	0.15

*CCD* ciliochoroidal detachment, *IOP* intraocular pressure, *NA* not applicable

## Discussion

In the present study, we prospectively investigated, by using swept-source AS-OCT, the presence of CCD before and after an 360S-LOT ab interno procedure, and we evaluated the effects of postoperative CCD on IOP for 12 postoperative months. We detected postoperative CCD in 21 eyes (47.7%) within postoperative day 7, and CCD was associated with lower IOP levels at only postoperative day 1. Higher CCD grades and wider CCDs were associated with lower IOP values within postoperative day 7. However, postoperative CCD had no effect on the postoperative IOP value after postoperative month 1 through postoperative month 12.

Akagi et al. reported that CCD occurred in 42% eyes on postoperative day 3 after ab interno trabeculotomy (with the Trabectome) [11]. Although we hypothesized that CCD may occur more frequently in eyes that had 360S-LOT performed than after Trabectome surgery because of the wider incision of Schlemm’s canal, the results showed that the presence and extent of CCD were almost the same for both surgical procedures. As Akagi et al. also mentioned, how the CCD occurred after the various surgeries is still unclear. Our study here found the following results: in 6 of 21 eyes in the CCD group, the anterior chamber connected to the CCD within postoperative day 7. In addition, in five of those six eyes, we observed the root of the iris after intraoperative peeling of the trabecular meshwork by suture or microhook needle. These findings suggested that the cyclodialysis cleft formed during 360S-LOT ab interno and enhanced the uveoscleral outflow, thereby causing transient CCD.

Postoperative hypotony is another possible cause of postoperative CCD. A reduced IOP reportedly induced CCD by allowing fluid to accumulate in the interstitial spaces and transudate through the choroidal capillary walls [16]. In the present study, we confirmed no wound leakage and an IOP value above 18 mm Hg at the end of surgery. Nevertheless, we cannot completely rule out the effect of transient hypotony on postoperative CCD, because self-sealing of the corneal incision is not always reliable after surgery.

Inflammation is an additional possible cause of postoperative CCD. Inflammation such as scleritis or Vogt–Koyanagi–Harada syndrome reportedly increased the permeability of the choroidal capillaries and induced CCD [16, 17]. Although we did not observe such a severe inflammation in the conjunctiva, sclera, anterior chamber, vitreous, or retina, inflammation induced by surgery cannot be ruled out completely as a cause of CCD. Thus, the mechanisms of the production of postoperative CCD are still unclear, but these different mechanisms may comprise a complicated mix and result in various types of CCD, such as CCDs with a different onset or extent.

The factors that affected the postoperative 12-month IOP were preoperative IOP and the combination procedure performed with cataract surgery. Outflow facility has been reported to be reduced in glaucomatous eyes because of to a decrease in Schlemm’s canal area and a reduction in the number of open collector channels available for fluid movement [18]. Such nonfunctional collector channels in eyes with high preoperative IOP may affect the risk for lower postoperative 12-month IOP. Phacoemulsification alone, however, can reduce the IOP in glaucomatous eyes [19, 20]. In the present study, we performed 360S-LOT with phacoemulsification in ~80% of eyes in both groups, and thus this high percentage (use of the combination procedure) may have affected the postoperative 12-month IOP.

The present study has certain limitations. The sample size was small and the follow-up period was 12 months, which was not long. The possibility exists that postoperative CCD may affect the IOP even after 12 postoperative months. However, all postoperative CCD disappeared within 2 months after surgery, and the IOP values were stable throughout the postoperative 1 to 12 months. Thus, CCD would probably not affect the postoperative IOP after 12 postoperative months. Additional investigations with larger sample sizes and longer follow-up periods are necessary to evaluate the importance of postoperative CCD and its long-term effects.

In conclusion, postoperative CCD occurred in approximately half of patients after 360S-LOT ab interno and may have the transient effect of lowering the IOP immediately after surgery but not the later IOP values until 12 months of follow-up.

### Summary

#### What was known before

- Ciliochoroidal detachment (CCD) sometimes occurred and might be associated with low intraocular pressure in the early postoperative period after several glaucoma surgeries. Whether CCD, however, affected mid- or long-term surgical results was still unclear.

#### What this study adds

- What this study adds Postoperative CCD occurred in approximately half of patients after 360-degree suture trabeculotomy ab interno and may have the transient effect of lowering the intraocular pressure immediately after surgery, but not the later intraocular pressure values until 12 months of follow-up.
